# Resection of ruptured aneurysm associated with bilateral anomalous posterior inferior cerebellar anastomotic arteries: case report and review of literature

**DOI:** 10.3389/fneur.2023.1281124

**Published:** 2023-12-01

**Authors:** Qingdong Han, Zongqi Wang, Tong Liu, Yabo Huang

**Affiliations:** ^1^Department of Neurosurgery, The First Affiliated Hospital of Soochow University, Suzhou, Jiangsu, China; ^2^Department of Pathology, Yantai Affiliated Hospital of Binzhou Medical College, Yantai, Shandong, China

**Keywords:** subarachnoid hemorrhage, anatomical variants, aneurysm, posterior inferior cerebellar artery anastomosis, surgical treatment

## Abstract

**Introduction:**

Aneurysms on the posterior inferior cerebellar artery (PICA) may not be the major part of intracranial aneurysm. Especially, an aneurysm located on the bilateral posterior inferior cerebellar anastomotic artery has abnormal anatomical characteristics in the vessel wall and then causes stroke including subarachnoid hemorrhage. This case report explores the direct resection of a ruptured aneurysm associated with the bilateral anomalous anastomotic artery of PICA.

**Methods:**

The case report discusses a 53-year-old woman who suffered from sudden severe headache and vomiting for more than 3 h admitted to our hospital. Emergency computed tomography (CT) revealed subarachnoid hemorrhage (SAH) in the third and fourth ventricles. Preoperative 3 Dimensions-digital subtraction angiography (3-D DSA) indicated a ruptured aneurysm located on the bilateral posterior inferior cerebellar anastomotic artery. Postoperative pathological findings indicated the characteristics of parent artery PICA and control aneurysm. The authors performed an overview of PICA aneurysms with anomalous variation in the Pubmed, Web of Science, and Medline databases. The search was until 1 August 2023. Related terms “posterior inferior cerebellar artery” And “aneurysm” AND “anatomical variants” were used to search the review. The reasons for anomalous variation anastomosis between bilateral PICAs were analyzed.

**Results:**

The aneurysm was resected successfully. Post-operative 3-D DSA revealed the disappearance of the aneurysm. The vessel wall of anastomotic PICA showed neovascularized hyperplasia, abnormal arrangement of smooth muscle, CD31+ endothelial cells, and SMA+ smooth muscle cells. In contrast, when it came to aneurysm, the wall at the location of the fracture thinned, which could be used to explain that the local nodular protrusion was formed and CD31+ endothelial cells existed. No neurological deficits were found at her 1-year follow-up visit (mRS score of 0).

**Conclusion:**

Direct resection of ruptured aneurysm associated with bilateral anomalous posterior inferior cerebellar anastomotic arteries was an effective treatment and careful consideration of the anatomical characteristics concerning the interesting aneurysm and the variant PICA was critical for sate treatment. Also, the literature on the lesion was reviewed.

## Introduction

Posterior inferior cerebellar artery aneurysms are rare and account for less than 3% of incidence (1). Due to its variants in the process of fetal development (2), arterial network concerning PICA indicates various pathological characteristics and may be an essential factor associated with some posterior circulation vascular diseases. Additionally, the mentioned features affect the collateral circulation and hemodynamics of PICA, causing aneurysms which can be a culprit of stroke. As to the PICA aneurysm with variant vessel, coiling, direct clipping, and trapping, the aneurysm with PICA-PICA anastomosis had different outcomes (1, 3–5). Especially, patients with PICA aneurysms suffered postoperative posterior ischemic stroke and nerve paresis, which makes the options concerning the variant PICA aneurysm unfavorable and negative.

The authors report one case of a ruptured aneurysm located on the bilateral posterior inferior cerebellar anastomotic artery. The ruptured aneurysm was resected leaving no neurological deficits, and further pathological characteristics between the variant PICA and control aneurysm were shown. In short, direct resection of the ruptured aneurysm was performed on the patient, allowing for the anatomical characteristics and imaging findings. To the best of our knowledge, the present study is the first research concerning direct resection of the aneurysm associated with the bilateral Anomalous Posterior Inferior Cerebellar Artery Anastomotic Arteries. Related literature on the lesion was reviewed and studied.

## Case description

**Figure 1:**
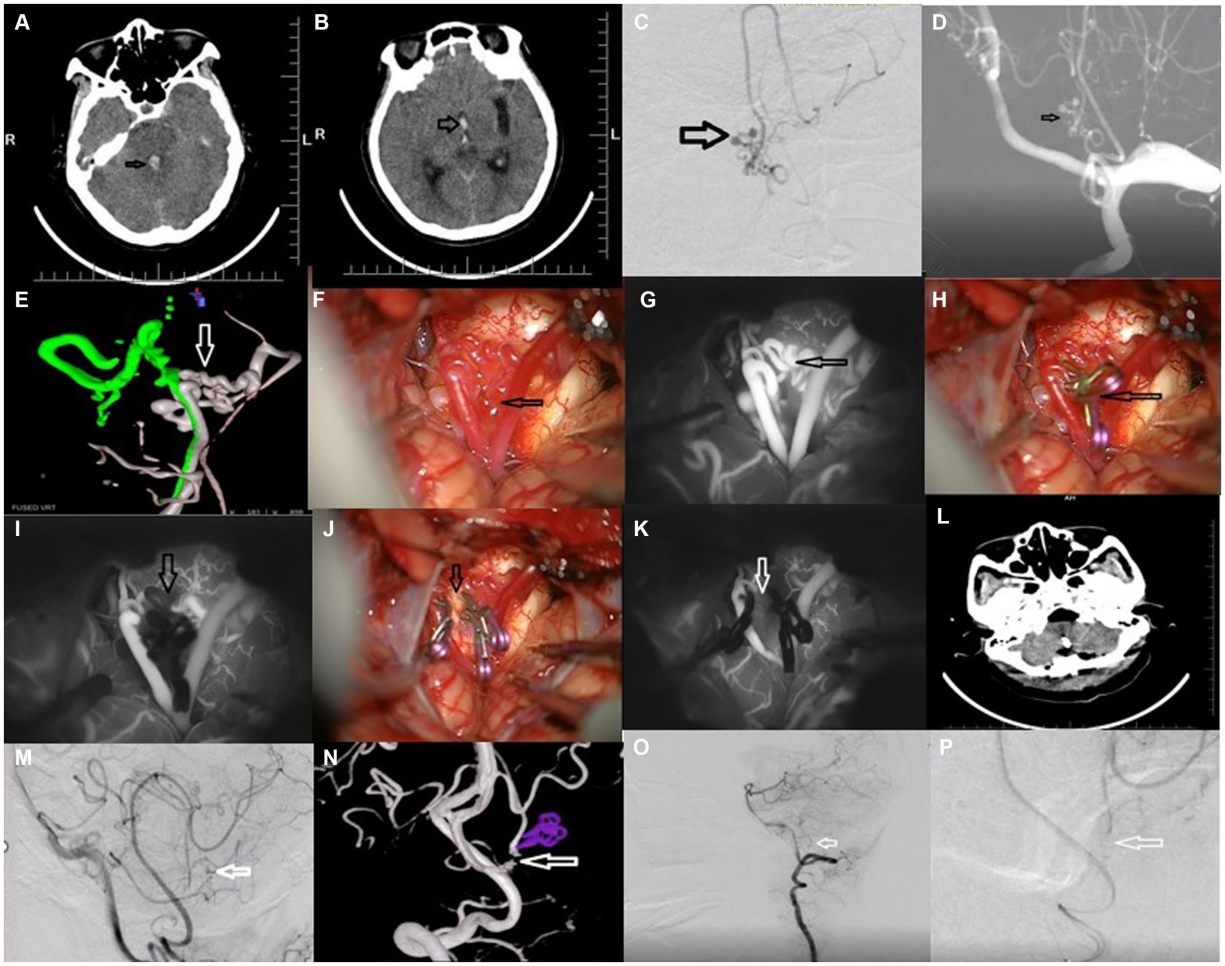
Resection of ruptured aneurysm associated with bilateral anomalous posterior inferior cerebellar anastomotic arteries.

A 53-year-old woman suffering from sudden severe headache and vomiting for more than 3 h was admitted to our hospital. Emergency CT revealed subarachnoid hemorrhage in the localization of the third and fourth ventricles (Figures 1A,B). Preoperative DSA and 3-D DSA indicated a ruptured aneurysm located on the bilateral posterior inferior cerebellar anastomotic artery (Figures 1C–E). The intraoperative image indicated an aneurysm located on the bilateral posterior inferior cerebellar anastomotic artery with some arterial branches on the back of the medulla (Figures 1F,G).

Concerning the anatomical characteristics and imaging findings, direct coiling was considered dangerous and may bring about ischemic events. Direct clipping of the aneurysm in the fourth ventricle may cause ischemic events and recurrence following clipping for anastomosis between bilateral PICAs of the anatomical characteristics. The woman was positioned in the right lateral park-bench position. Suboccipital craniotomy in the midline was performed. The aneurysm was exposed by a suboccipital approach in the midline. Bilateral posterior inferior cerebellar arteries were trapped prior to resection of the ruptured PICA aneurysm (Figures 1H,I). After trapping the anastomosis between bilateral PICAs, the aneurysm was resected successfully and intraoperative ICG showed the disappearance of the aneurysm (Figures 1J,K). Post-operative CT (Figure 1L), post-operative DSA, and 3-D DSA revealed the obliteration of the aneurysm (Figures 1M–P). Postoperative pathological findings indicated the characteristics of the variant aneurysm. The woman was discharged on the seventh postoperative day (mRS score of 0) and no neurological deficits were found at her 1-year follow-up visit.

## Pathological characteristics

An in-depth study of the histopathological findings was conducted for the treatment of the disease which helped the authors understand the pathogenic mechanism.

Postoperative pathological findings indicated significant differences between the present variant posterior inferior cerebellar anastomotic artery (V-PICA) and another aneurysm (the clinical features of another patient just suffering an aneurysm that was not mentioned in the present study).

### Variant posterior inferior cerebellar anastomotic artery

**Figure 2:**
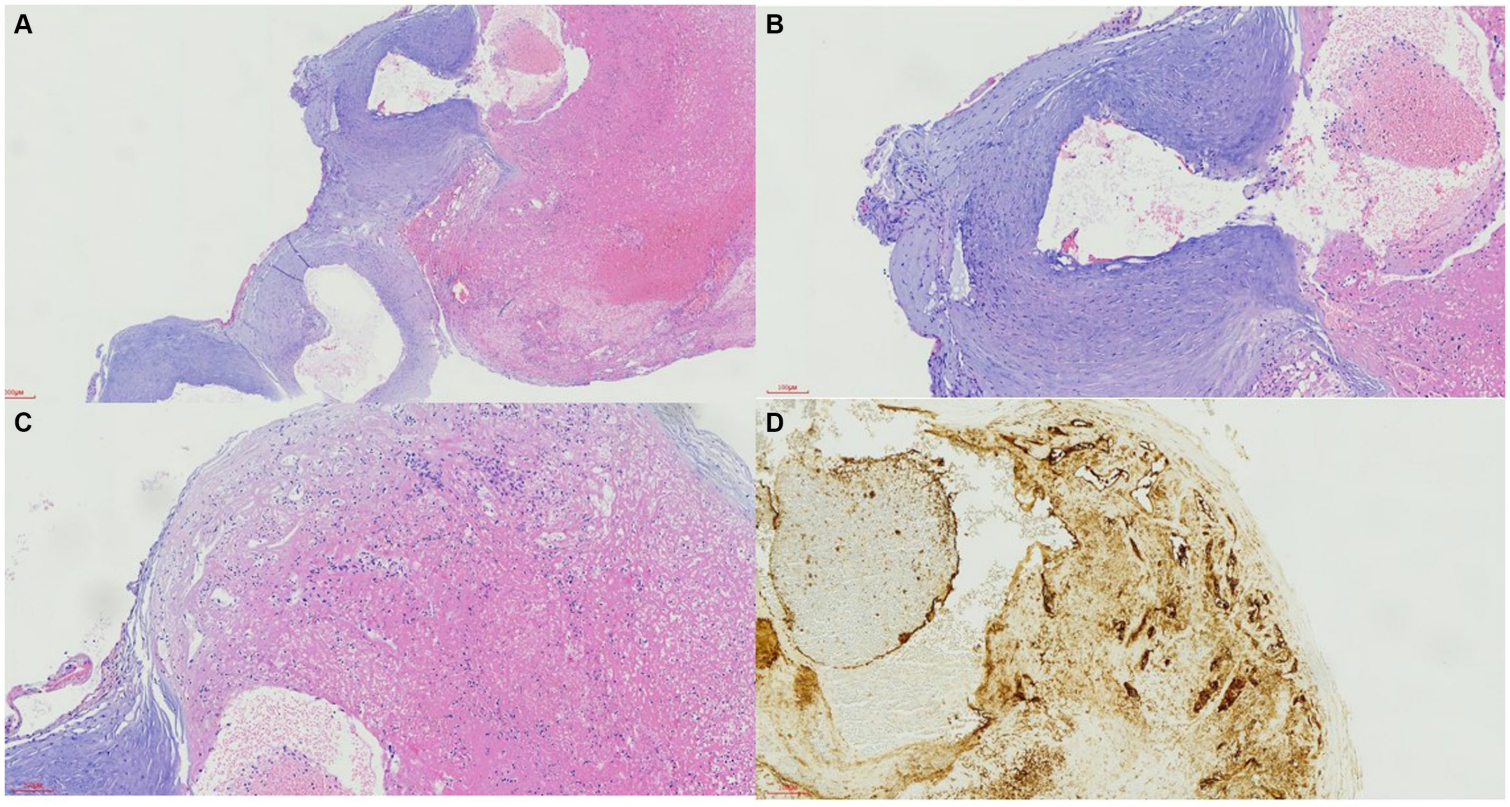
Pathological findings in the vessel wall of variant posterior inferior cerebellar anastomotic artery.

HE staining (Figure 2A) showed that the vessels were twisted, with uneven thickness of the vessel wall, obvious thickening of part of the vessel wall, and rupture of part of the vessel wall with bleeding. Mechanized neovascularization and hyperplasia and lumen sizes were observed at remote bleeding sites (original magnification ×40).

HE staining (Figure 2B) revealed that the smooth muscle of the malformed vessel wall was disordered, the lumen of the ruptured vessel was connected with the remote mechanized hemorrhage, and vascular endothelial cell proliferation was observed (original magnification ×100).

HE staining (Figure 2C) indicated that the partial vascular wall was disordered with remote hemorrhage accompanied by thrombosis, fibrin aggregation, and mechanical angiogenesis with lumen varying in size, and vascular endothelial cell proliferation could be seen in some thrombotic areas (original magnification ×100).

CD31 immunohistochemical staining (Figure 2D, original magnification ×100) revealed a positive expression of vascular endothelial cells, vascular lumen disorder, and some new vessels in the vascular wall along the original vessel wall, and the lumen size was different, which shows mechanical thrombosis.

### Aneurysm

**Figure 3:**
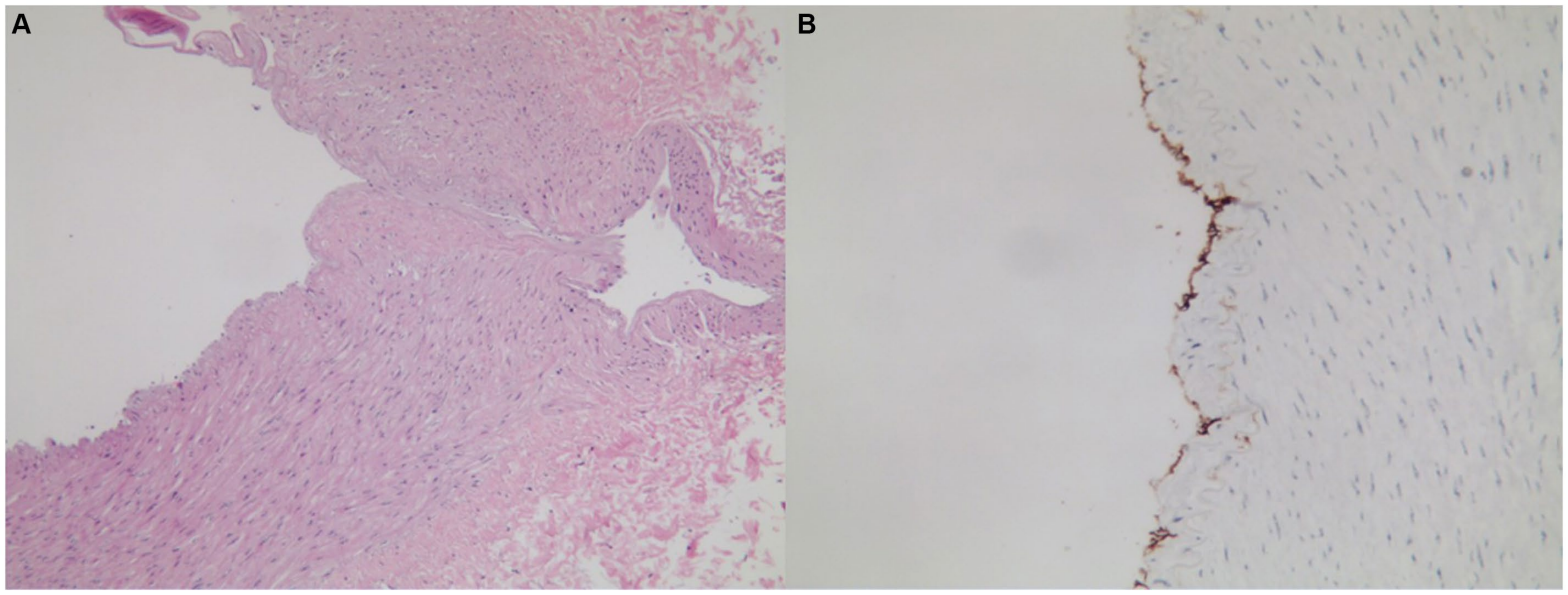
Pathological findings in the vessel wall of control aneurysm.

HE staining (Figure 3A, original magnification ×40) showed that the arterial vessel wall was broken, the vascular endothelium extended outside the vessel wall along the fracture site, and the bleeding at the fracture site was significantly thinned. It could be found that local nodular protrusion formed when the vessels were in the filling state.

CD31 immunohistochemical staining indicated positive staining for vascular endothelial cells (Figure 3B, original magnification ×100).

## Literature search and analysis

The authors performed an overview of PICA aneurysms with anomalous variation in the Pubmed, Web of Science, and Medline databases. The search was until 1 August 2023.

Related terms “posterior inferior cerebellar artery” And” aneurysm” AND “anatomical variants” AND “English” were used to search the databases. The authors followed the PRISMA guideline previously reported and manually extracted relevant references. Furthermore, all searched articles were carefully checked and reviewed (Table 1). In eight reviewed articles, different patients suffered SAH, headache, intra-ventricular hemorrhage, obtundation, quadriplegia, giddiness, nausea, vertigo, and right hypoacusis. The ages of patients ranged from 19 to 78 years. Endovascular treatment, direct clipping of PICA-PICA anastomosis combined with trapping of the related PICA aneurysm, coil embolization of the aneurysm, and further parent vessel occlusion and posterior fossa decompression by suboccipital craniectomy for rebleeding, reimplantation bypass, and trapping of the aneurysm were taken into consideration. Various neurological statuses at discharge and follow-up are shown in Table 1.

**Table 1:** Overview of PICA aneurysm with anomalous variation.

References	Sex, age	Clinical sign	Diseased region	Treatment	Neurological status at discharge	Follow-up
Joseph et al. (6)	19, male	SAH	AICA and bulbar artery supply to PICA	Coil embolization of the An	Left Horner, s syndrome	4 months
Fujimura et al. (2)	50, male	Headache, obtundation, and quadriplegia	PICA-AICA anastomotic Artery	Coil embolization of An and occlusion of the PA	moderate disturbance of consciousness and quadriparesis	-
Baskaya et al. (7)	44, female	SAH and IVH	AICA-PICA anastomotic Artery	Clipping	Hydrocephalus and lower cranial nerve paresis	3 years
Chin et al. (3)	54, male	Cerebellar hematoma and IVH	Bihemispheric PICA	Coil embolization of An, and further PVO and posterior fossa decompression by suboccipital craniectomy for rebleeding	mRS 1	6 months
Dammers et al. (8)	52, female	SAH	Extracranial PICA	Clipping	No major neurological deficits	7 days
Germans et al. (9)	49，male	SAH	LSA-PICA	Clipping	Uneventful	6 months
Lang et al. (4)	78, male	SAH	Double-origin PICA	Reimplantation bypass and trapping of the aneurysm	Glasgow coma scale score of 15 and mRS 3	5 months
Jerry et al. (5)	39, male	Vertigo and right hypoacusis	AICA-PICA common trunk variant	Endovascular treatment	Intact neurological status	7 months
Present study	53, female	SAH and IVH	Bilateral PICA anastomosis	Resection of the An	mRS 0	1 year

PICA, Posterior inferior cerebellar artery; An, Aneurysm; PA, Parent artery; IVH, Intraventricular hemorrhage; mRS, modified Rankin score; PVO, Parent vessel occlusion.

### Follow-up

The patient returned to normal life without postoperative complications. In addition, no neurological disturbances occurred at 1-year follow-up (mRS score of 0).

## Discussion

Although dominantly originating from the vertebral artery, it is well known that PICA has various variations. The variations may be responsible for some intracranial vascular diseases (3–5), including aneurysms. There were various clinical features of the V-PICA aneurysm. In the eight reviewed articles (Table 1), the symptoms included SAH, headache, intra-ventricular hemorrhage, obtundation, quadriplegia, giddiness, nausea, vertigo, and right hypoacusis (2–9). In our present case, the woman suffered a sudden severe headache and vomiting for more than 3 h before being admitted to our hospital, and preoperative CT indicated SAH.

Some embryonic arterial characteristics concerning V-PICA can be utilized to explain the aforementioned intracranial vascular diseases, especially PICA aneurysms. Usually, in the first 28 days, fetal development occurs in neural arteries and then in lateral vertebrobasilar anastomosis at 29 days (2). At that time, bilateral PICA had anastomosis and collateralization, and then the above anastomosis regresses. Additionally, the fetal anastomosis developed into the independent PICAs in most individuals (1). However, the other few individuals continued to have the features of fetal anastomosis and were unable to regress. Additionally, anastomosis and collateralization became anomalous and fragile (6), which was related to hemodynamic disturbance, then the hemodynamic stress owing to the mentioned anastomosis and collateralization led to vascular disease.

In the present case, the authors indicated that due to anomalous variation anastomosis between bilateral PICAs, the bilateral PICA suffered hemodynamic stress. The left PICA was dominant and the right PICA was non-dominant (Figure 1G). Due to the fragile anastomosis and collateralization between the two PICAs, the aneurysm occurred and was prone to rupture. Interestingly, the ruptured PICA aneurysm was located at the anastomosis and collateralization, which was similar to the formation of the anterior communicating artery aneurysm with one side having a dominant A1 segment and the other side having a non-dominant A1 segment. At the same time, the pathological findings revealed the differences between the present V-PICA and the control aneurysm (Figures 2, 3). In all, the anomalous anastomosis and collateralization were the culprits of the formation and rupture of the present aneurysm at the bilateral PICA anastomosis.

The pathological or etiology characteristics of V-PICA are dramatically different from those of other intracranial vascular lesions. In the present study, we found some interesting characteristics associated with the vessel wall in V-PICA and aneurysm. The vessel wall of V-PICA showed neovascularized hyperplasia, abnormal arrangement of smooth muscle, CD31+ endothelial cells, and SMA+ smooth muscle cells. In contrast, when it came to aneurysm, the wall at the location of the fracture thinned, which could be used to explain that the local nodular protrusion was formed and CD31+ endothelial cells existed.

Why are the treatments for V-PICA aneurysm dramatically different? In fact, because of a lack of a full understanding of the clinical features, the optimal strategy associated with V-PICA aneurysms is in debate. However, when it comes to the effective intervention for V-PICA aneurysms, various aspects are feasible and available. Previous studies show that endovascular treatment should be recommended as an ideal option following careful consideration of vascular features preoperatively, including sacrificing the parent artery PICA and its collateralization (2, 3, 5, 6). Directly clipping the ruptured V-PICA aneurysm can be performed in some corresponding patients, who may have higher postoperative complications (7–9). Even reimplantation bypass and trapping of the aneurysm may be done to prevent postoperative posterior circulation stroke (4). Compared with a perforating artery, anatomical variations and surgical access may give rise to sooner difficulties in treating PICA aneurysms, except that a perforating artery has a rich anastomotic network (10). PICA anastomotic arteries or the double origin of the two sides of PICAs had anomalous characteristics concerning anatomy. In the present study, we performed complete resection of the ruptured PICA aneurysm without postoperative neurological dysfunctions. To the best of our knowledge, the present study is the first study concerning direct resection of the V-PICA aneurysm.

However, more attention should be paid to some items. Firstly, preoperative analysis of the anatomical variations in PICA should be performed carefully. Secondly, the experience is limited for the present case and further studies concerning the lesion should be done for proper clinical options. Thirdly, other interventions can also be taken into consideration for mitigating the risk.

## Conclusion

Direct resection of ruptured aneurysm associated with bilateral anomalous posterior inferior cerebellar anastomotic arteries was an effective treatment, and careful consideration of the anatomical characteristics concerning the interesting aneurysm and the variant PICA was critical for sate treatment.

## Data availability statement

The datasets presented in this article are not readily available because of ethical and privacy restrictions. Requests to access the datasets should be directed to the corresponding author.

## Ethics statement

The studies involving humans were approved by the Ethics Committee of the First Affiliated Hospital of Soochow University. The studies were conducted in accordance with the local legislation and institutional requirements. The participants provided their written informed consent to participate in this study. Written informed consent was obtained from the individual(s) for the publication of any potentially identifiable images or data included in this article.

## Author contributions

QH: Resources, Writing – original draft, Writing – review & editing. ZW: Writing – original draft. TL: Data curation, Resource, Writing – original draft. YH: Resources, Supervision, Writing – original draft, Writing – review & editing.
